# Supplementary material for: Evaluating the Prognostic Value of the Triglyceride–Glucose Index in Different Populations: A Critical Analysis
Source: Nutrients. 2025 Mar 24;17(7):1124. doi: 10.3390/nu17071124 (PMC11990857; doi:10.3390/nu17071124)
Supplement: Supplementary file 1 [file nutrients-17-01124-s001.zip › nutrients-3502454-supplementary.pdf]

nutrients MDPI

Supplementary Materials

The Supplementary shows the prognostic value of individual factors at Cox proportional analysis calculated in studies in obese subjects (Supplementary Table S1, Cohort 1) and various Cox regression models calculated in Cohort 1 (Supplementary Table S2, A to F) and in a fraction or Cohort 2 (General population cohort, subjects with BMI > 30 kg/m^2, Supplementary Table S2 G). Details of abbreviations and of reference to the Charlson Comorbidity index are reported after Table S2 G).

Supplementary Table S1. Prognostic value of individual factors at Cox proportional analysis in studies in obese subjects. HR, p value, and Harrell's C Index are shown for each factor.

	Study in obese subjects		
**Factor**	**HR**	*p*	Harrell (%)
Age	1.082	< 0.001	73.7
Female Sex	0.560	< 0.001	56.2
Charlson Index * §	3.126	< 0.001	68.8
Metabolic syndrome *	1.692	< 0.001	64.5
Diabetes	3.027	< 0.001	62.8
GT *	1.957	< 0.001	65.4
BGQ	1.808	< 0.001	67.1
TYGQ	1.621	< 0.001	64.4

* Calculated only in cohort 1

Supplementary Table S2. Cox regression models.

A. Obese cohort. Glucose Tolerance-based models.

	**Basic model**	**Model 1**	**Model 2**	**Model 3**
**Variables (units of increase)**	**HR (95% C.I.), p-value**	**HR (95% C.I.), p-value**	**HR (95% C.I.), p-value**	**HR (95% C.I.), p-value**
Age (years)	1.076 (1.059-1.095), p<0.001	1.075 (1.057-1.093), p<0.001	1.077 (1.059-1.095), p<0.001	1.076 (1.057-1.094), p<0.001
Sex (females versus males)	0.538 (0.387-0.748), p<0.001	0.535 (0.385-0.744), p<0.001	0.562 (0.404-0.783), p=0.001	0.558 (0.401-0.778), p=0.001
**Glucose Tolerance**	1.408 (1.154-1.717), p=0.001	1.016 (0.613-1.685), p=0.950	1.228 (0.970-1.554), p=0.088	0.973 (0.581-1.631), p=0.919
BGQ		1.348 (0.881-2.061), p=0.168		1.251 (0.807-1.938), p=0.316
TYGQ			1.218 (1.008-1.471), p=0.041	1.191 (0.982-1.446), p=0.076
Harrell's C index	*76.1%*	*76.3%*	*76.4%*	*76.5%*

B. Obese cohort. Metabolic syndrome-based models.

	**Basic model**	**Model 1**	**Model 2**	**Model 3**
**Variables (units of increase)**	**HR (95% C.I.), p-value**	**HR (95% C.I.), p-value**	**HR (95% C.I.), p-value**	**HR (95% C.I.), p-value**
Age (years)	1.078 (1.060-1.095), p<0.001	1.074 (1.056-1.093), p<0.001	1.078 (1.060-1.096), p<0.001	1.075 (1.057-1.093), p<0.001
Sex (females versus males)	0.524 (0.378-0.726), p<0.001	0.540 (0.389-0.750), p<0.001	0.552 (0.396-0.769), p<0.001	0.555 (0.399-0.774), p<0.001
**Metabolic syndrome**	1.347 (1.131-1.604), p=0.001	1.191 (0.970-1.461), p=0.095	1.169 (0.926-1.475), p= 0.190	1.112 (0.874-1.414), p=0.388
BGQ		1.251(1.028-1.522), p=0.025		1.201(0.975-1.479), p=0.084
TYGQ			1.212 (0.980-1.499), p=0.076	1.1294 (0.90-1.416), p=0.291
Harrell's C index	*76.1%*	*76.5%*	*76.3%*	*76.5%*

C. Obese cohort. Arterial Hypertension-based models.

	**Basic model**	**Model 1**	**Model 2**	**Model 3**
**Variables (units of increase)**	**HR (95% C.I.), p-value**	**HR (95% C.I.), p-value**	**HR (95% C.I.), p-value**	**HR (95% C.I.), p-value**
Age (years)	1.078 (1.060-1.096), p<0.001	1.072 (1.053-1.090), p<0.001	1.075 (1.057-1.094), p<0.001	1.072 (1.054-1.091), p<0.001
Sex (females versus males)	0.489 (0.354-0.677), p<0.001	0.529 (0.381-0.735), p<0.001	0.547 (0.393-0.763), p<0.001	0.552 (0.396-0.769), p<0.001
**Arterial Hypertension**	1.507 (1.087-2.089), p=0.014	1.348 (0.967-1.881), p=0.078	1.367 (0.980-1.906), p=0.066	1.316 (0.941-1.839), p=0.108
BGQ		1.327 (1.118-1.575), p=0.001		1.203 (0.980-1.477), p=0.077
TYGQ			1.299 (1.105-1.527), p=0.002	1.174 (0.967-1.426), p=0.105
Harrell's C index	*75.9%*	*76.8%*	*76.6%*	*76.9%*

D. Obese cohort. Coronary Heart Disease -based models.

	**Basic Model**	**Model 1**	**Model 2**	**Model 3**
**Variables (units of increase)**	**HR (95% C.I.), p-value**	**HR (95% C.I.), p-value**	**HR (95% C.I.), p-value**	**HR (95% C.I.), p-value**
Age (years)	1.081 (1.064-1.091), p<0.001	1.073 (1.055-1.091), p<0.001	1.077 (1.060-1.095), p<0.001	1.073 (1.053-1.092), p<0.001
Sex (females versus males)	0.493 (0.356-0.683), p<0.001	0.529 (0.381-0.735), p<0.001	0.553 (0.397-0.770), p<0.001	0.553 (0.397-0.771), p<0.001
**Coronary Heart Disease**	1.739 (1.013-2.983), p=0.045	1.745 (1.018-2.992), p=0.043	1.654 (0.963-2.842), p=0.068	1.702 (0.991-2.923), p=0.054
BGQ		1.367 (1.154-1.618), p<0.001		1.235 (1.009-1.512), p=0.041
TYGQ			1.325 (1.130-1.554), p=0.001	1.179 (0.973-1.428), p=0.092
Harrell's C index	*75.2%*	*76.4%*	*76.3%*	*76.6%*

E. Obese cohort. Charlson Comorbidity index-based models.

	**Basic model**	**Model 1**	**Model 2**	**Model 3**
**Variables (units of increase)**	**HR (95% C.I.), p-value**	**HR (95% C.I.), p-value**	**HR (95% C.I.), p-value**	**HR (95% C.I.), p-value**
Age (years)	1.064 (1.044-1.085), p<0.001	1.064 (1.043-1.086), p<0.001	1.066 (1.045-1.088), p<0.001	1.066 (1.044-1.088), p<0.001
Sex (females versus males)	0.509 (0.367-0.705), p<0.001	0.537 (0.386-0.745), p<0.001	0.558 (0.400-0.777), p=0.001	0.559 (0.401-0.778), p= 0.001
**Charlson Comorbidity Index §**	1.615 (1.143-2.279), p=0.006	1.358 (0.943-1.956), p=0.101	1.424 (0.999-2.029), p= 0.051	1.328 (0.920-1.916), p=0.130
BGQ		1.295 (1.086-1.544), p=0.004		1.176 (0.956 -1.446), p=0.125
TYGQ			1.279 (1.087-1.507), p=0.003	1.177 (0.969-1.428), p=0.099
Harrell's C index	*75.6%*	*76.5%*	*76.4%*	*76.6%*

F. Obese cohort. Blood glucose-based models, with blood glucose expressed as quartiles (BGQ).

	**Basic model**	**Model 1**	**Model 2**
**Variables (units of increase)**	**HR (95% C.I.), p-value**	**HR (95% C.I.), p-value**	**HR (95% C.I.), p-value**
Age (years)	1.075 (1.057-1.093), p<0.001	1.079 (1.062-1.098), p<0.001	1.075 (1.057-1.094), p<0.001
Sex (females versus males)	0.541(0.390 -0.752), p<0.001	0.562 (0.403-0.781), p=0.001	0.565 (0.406-0.787), p=0.001
**BGQ**	1.361 (1.150-1.611), p<0.001		1.217 (0.995-1.489), p=0.056
TYGQ		1.338 (1.141-1.570), p<0.001	1.200 (0.991-1.455), p=0.062
Harrell's C index	0.7625	0.7609	0.7640

G. General population cohort (subjects with BMI > 30 kg/m2). Diabetes based models

	**Basic model**	**Model 1**	**Model 2**	**Model 3**
**Variables (units of increase)**	**HR (95% C.I.), p-value**	**HR (95% C.I.), p-value**	**HR (95% C.I.), p-value**	
Age (years)	1.09 (1.08 – 1.10), p<0.001	1.09 (1.08 – 1.10), p<0.001	1.09 (1.08 – 1.10), p<0.001	1.09 (1.08 – 1.10), p<0.001
Sex (females versus males)	0.64 (0.56 – 0.72), p<0.001	0.63 (0.56 – 0.72), p<0.001	0.63 (0.56 – 0.72), p<0.001	0.63 (0.56 – 0.72), p<0.001
**Diabetes**	1.61 (1.41 – 1.83), p<0.001	1.63 (1.41 – 1.88), p<0.001	1.64 (1.41 – 1.88), p<0.001	1.65 (1.42 – 1.91), p<0.001
BGQ		0.98 (0.91 – 1.06), p=0.633		1.00 (0.92-1.08), p=0.943
TYGQ			0.95 (0.88 – 1.02), p=0.147	0.95 (0.87-1.02), p=0.171
Harrell's C index	*83.6%*	*83.6%*	*83.7%*	*83.7%*

BG = blood glucose; TYG = triglyceride–glucose index; * TYG = ln [triglycerides (mg/dl) x blood glucose (mg/dl)/2]; BGQ = blood glucose quartiles; TYGQ = triglyceride–glucose index Quartiles; GT = Glucose tolerance. § Charlson ME, Pompei P, Ales KL, MacKenzie CR. A new method of classifying prognostic comorbidity in longitudinal studies: development and validation. J Chronic Dis. 1987; 40: 373-383.
